# A preliminary analysis of “Passport to practice”: investigating development of core competencies in undergraduate health promotion students

**DOI:** 10.1177/17579759241230065

**Published:** 2024-04-01

**Authors:** Krysten Blackford, Malena Della Bona, Gemma Crawford

**Affiliations:** School of Population Health, Curtin University, Perth, Australia

**Keywords:** capacity building, education, health promotion, workforce development, public health, evaluation, qualitative, quantitative

## Abstract

**Background:**

Equipping tertiary health promotion students with skills and knowledge to contribute meaningfully to the health promotion workforce begins with enhancing their health promotion competence via well-designed curriculum. This includes a focus on work-integrated learning, global citizenship, professional identity and competency mapping in line with the International Union for Health Promotion and Education Core Competencies and Professional Standards for Health Promotion.

**Methods:**

In this paper we report baseline results for the Passport to Practice project, a mixed-methods prospective cohort study to track undergraduate health promotion student progress across their degree, to evaluate a new approach for assessing student achievement of the Competencies and Standards developed by the International Union for Health Promotion and Education. Baseline data were collected from first-year students via document analysis of student reflection papers (*n* = 40); and an online survey (*n* = 29) to measure self-reported health promotion competence, development of global citizenship and professional identity, and PebblePad usability.

**Results:**

Findings suggest the Passport to Practice initiative positively contributed to professional identity and health promotion competence. Students appreciated work-integrated learning opportunities that enabled them to plan for future activities to address gaps in their competence; and students excelled in the social responsibility dimension of global citizenship but lagged in the political voice category of the global civic engagement dimension.

**Conclusion:**

Findings provide insights about strategies and concepts required to equip students with the skills and knowledge required for their role as health promotion practitioners to address complex public health challenges.

## Introduction

The development and application of core competencies to support health promotion university education, practitioners and organizations have proliferated over the past four decades (1). Core competencies for health promotion are generally agreed as the ‘the minimum set of competencies that constitute a common baseline for all health promotion roles’ (2, p.3). Recently, health promotion has been strengthened as a discipline and profession via the International Union for Health Promotion and Education (IUHPE) Health Promotion Accreditation System (3), which provides educational accreditation and practitioner registration based upon agreed criteria. The IUHPE Core Competencies and Professional Standards for Health Promotion (4) (Competencies and Standards herein) underpin the curriculum of courses accredited by IUHPE and are used by health promotion practitioners globally. The Competencies and Standards include nine domains (Enable Change; Advocate for Health; Mediate through Partnership; Communication; Leadership; Assessment; Planning; Implementation; Evaluation and Research) underpinned by ethical values and knowledge.

Curtin University’s Bachelor of Science (BSc) in Health Promotion was the first Australian course (degree/programme of study) to receive IUHPE accreditation (3). The three-year undergraduate course was established in 1987 and teaching staff have historically used competencies to guide course refinement and capture theory-to-practice knowledge transfer (5). Various methods have been used, including an evidence guide (6) or portfolio (5) to map competency development; reflective writing via an e-portfolio (7); and a long-running ‘Passport to Practice’ incorporating activities based on work-integrated learning (WIL) principles (8). In 2019, the course underwent renewal to prepare for re-accreditation by revising alignment with the Competencies and Standards (9). Areas identified for improvement were global citizenship, embedded practical activities, and professional identity.

Preparing students for health promotion practice includes developing their capacity to contribute meaningfully as global citizens (10). Global Citizenship Education (11) embeds global orientation and citizenship concepts and assessments into the curriculum (12). Dimensions of global citizenship are coterminous with health promotion competencies, specifically: social responsibility, global competence and global civic engagement (13). Good curriculum design focusing on global citizenship can enhance the likelihood that students will graduate with necessary skills to address complex public health challenges (10).

Professional identity is vital to how health promotion is understood, practised and advanced as a discipline and profession (11). Professional identity has been defined as ‘the formation of an attitude of personal responsibility regarding one’s role in the profession, a commitment to behave ethically and morally, and the development of feelings of pride for the profession’ (14, p.686). WIL and theory-to-practice approaches contribute to professional identity (15). Biehl and colleagues (16) have argued that professional identity formation can support professional roles in health promotion and that universities play a vital role in this.

To support enhanced WIL, professional identity and competency development, Passport to Practice was embedded across the course in 2021 (previously described in detail (9)). Briefly, the assessment series links three units (single subjects within a course (degree)) providing students with WIL opportunities and authentic assessments aligned with the Competencies and Standards and a focus on professional identity (17). Students map skills and knowledge against the Competencies and Standards in each unit, and participate in and reflect on practical co-curricular activities to emulate continuing professional development requirements for practitioner registration (18). Assessment is supported by PebblePad, an online platform used as a competency-based portfolio system for evidencing student achievements, experiences and skills (19).

The Passport to Practice research project aims to evaluate the new approach for assessing student achievement of the Competencies and Standards by following student progression across three years. This research has the following objectives: (i) examine student development of perceptions, awareness, knowledge and skills toward the Competencies and Standards; (ii) measure student development of global citizenship and professional identity; (iii) evaluate the utility of PebblePad; and (iv) provide a framework for undergraduate teaching and assessing the Competencies and Standards. This paper is a report of the baseline methods and results.

## Methods

In this section we outline the methods for year 1 of a mixed-methods prospective cohort study (Table 1), led by two teaching and research academics involved in designing and delivering the BSc in Health Promotion. Units that are part of the study are:

-  HLPR1000 Health Promotion Principles and Values: first-year unit introducing students to the principles and values of health promotion as a theoretical foundation for contemporary practice, policy and research;
-  HLPR2000 Health Promotion in Action: second-year unit providing a practical introduction to the design, implementation and evaluation of health promotion interventions;
-  HLPR3002 Health Promotion Leadership and Identity: third-year unit providing a professional capstone to synthesize degree theoretical and practical content.

**Table 1.** Study design.

Data type	Year 1 (2021 + 2022)	Year 2 (2022 + 2023)	Year 3 (2023 + 2024)
**Qualitative**	• HLPR1000 document analysis	• HLPR2000 document analysis • Student focus groups	• HLPR3002 document analysis • Student focus groups • Staff focus group
**Quantitative**	• Student survey	• Student survey	• Student survey

## Participant recruitment

Students commencing their studies in 2021 and 2022 in a single or double (Health and Safety or Nutrition) Health Promotion or Health Sciences (incorporating Health Promotion units) undergraduate degree and enrolled in HLPR1000 (2021: *n* = 53; 2022: *n* = 64) were invited to participate in year 1 of the study.

## Data collection

### Student survey

Students were invited to complete an online survey utilizing Qualtrics via announcements posted in the learning management system. Questions collected demographic information and enrolment status (adapted from Wold *et al.* (20)); health promotion competence; global citizenship; professional identity; and PebblePad usability. Psychometrics of the adapted instruments utilized were not rechecked because of previous validity and reliability testing, as described below. Additionally, the instrument was piloted with third-year health promotion students to enhance face and content validity (21).

### Health promotion competence

Students self-rated their current knowledge and skills in the Competencies and Standards by indicating their level of agreement using five-point Likert scales (strongly agree to strongly disagree) against statements comprising descriptions of each domain. For example, ‘I have the knowledge and skills required to enable change (domain 1): “Enable individuals, groups, communities and organizations to build capacity for health promoting action to improve health and reduce health inequities.”’ Open-ended questions asked students to describe units and practical activities contributing to their knowledge and skill development.

### Global citizenship

Items were included from the Global Citizenship Scale, which has been tested for face validity, exploratory and confirmatory factor analyses, and nominal group technique to verify the scope of the global citizenship construct (13). Students indicated their level of agreement with 28 statements using five-point Likert scales (strongly disagree to strongly agree) across three dimensions: social responsibility (global justice and disparities, altruism and empathy, global interconnectedness and personal responsibility); global competence (self-awareness, intercultural communication, global knowledge); and global civic engagement (involvement in civic organizations, political voice, global civic activism). Two items were removed from the global civic engagement section which related to short-term overseas travel plans due to international border closures in Australia during the COVID-19 pandemic.

### Professional identity

The Macleod Clark Professional Identity Scale (22) was used to measure professional identity, which demonstrated predictive validity among undergraduate nursing students. The instrument items were adapted to include the term ‘health promotion’ as the profession and nine statements exploring feelings of belonging to and positively identifying with the health promotion profession were included. Students indicated their level of agreement with the statements using five-point Likert scales (strongly disagree to strongly agree). For example, ‘I feel I have strong ties with members of the health promotion profession’. Each item was scored between 1 and 5 to provide a total score between 9 and 45 across nine items. Higher scores indicate higher self-reported professional identity.

### PebblePad usability

PebblePad usability was assessed using the System Usability Scale (SUS) (23), a 10-item instrument used to test online platforms using five-point Likert scales (strongly disagree to strongly agree). For example, ‘I found the various functions in PebblePad were well integrated’. The SUS has demonstrated excellent reliability (coefficient alpha > 0.90), validity and sensitivity for measuring usability (24). Open-ended questions provided additional comments regarding PebblePad use.

## Document analysis

All students enrolled in HLPR1000 submit reflection papers as part of an assessment to map competency development. Students were invited to provide consent for their reflection to be included in the study after the assessment had been submitted and graded, and marks released. Identifiable information was removed by researchers after document extraction.

## Data analysis

Quantitative data were analysed in SPSS (25) and descriptive statistics were generated. The SUS scoring protocol was implemented for PebblePad usability data, whereby item scores were summed for each participant, out of 100. The average score was calculated and categorized as follows: ⩾80 = A; 70–79 = B; 60–69 = C; 50–59 = D; and ⩽50 = F. Mean scores were calculated for Likert scale items in the Global Citizenship Scale and the Macleod Clark Professional Identity Scale (1 = strongly disagree, 3 = neutral, 5 = strongly agree). Mean scores for negatively worded items were calculated using inverse scale values. Qualitative data were managed using NVivo (26) and directed content analysis was conducted (27) using the Competencies and Standards as a framework. Data triangulation was carried out to corroborate the qualitative and quantitative results (28).

## Ethical considerations

The Curtin University Human Research Ethics Committee approved the study (HRE2021-0169). Participants provided informed consent and students were advised that non-participation would not affect their enrolment status. Because this research involved university teaching staff, all data were initially analysed by an independent Project Officer. Results were reviewed by primary researchers.

## Results

### Survey sample

Table 2 summarizes baseline demographic characteristics, comprising 10 students in 2021 (18.9% response) and 19 students in 2022 (29.7% response). The majority were women (*n* = 27, 93.1%), born in Australia (*n* = 17, 58.6%), in 2000 or later (*n* = 13, 44.8%), grew up in a major city (*n* = 19, 65.5%), living with their parents (*n* = 16, 55.2%), domestic students (*n*=28, 96.6%), enrolled in the BSc Health Promotion (*n* = 16, 55.2%) and studying full time (*n* = 15, 51.7%). Because of the small sample size, differences between demographic groups were not analysed for the survey results.

**Table 2.** Baseline survey sample demographic characteristics (*N* = 29).

Variable	*n* (%)
Year of birth	
1970–1979	5 (17.2)
1980–1989	3 (10.3)
1990–1999	8 (27.6)
2000 onwards	13 (44.8)
Gender	
Man	2 (6.9)
Woman	27 (93.1)
Location growing up	
Regional	10 (34.5)
Major city	19 (65.5)
Country of birth	
Australia	17 (58.6)
Other	12 (41.4)
Living arrangements	
Parent’s home	16 (55.2)
Share house	1 (3.4)
Own home	8 (27.6)
Partner’s home	3 (10.3)
Other	1 (3.4)
Student type	
Domestic	28 (96.6)
International	1 (3.4)
Course	
BSc Health Promotion	16 (55.2)
BSc Health Promotion, BSc Health and Safety	1 (3.4)
BSc Nutrition, BSc Health Promotion	6 (20.7)
BSc Health Sciences	6 (20.7)
Enrolment status	
Full time	15 (51.7)
Part time	14 (48.3)

### Health promotion competence

Table 3 summarizes baseline health promotion competence. Students rated themselves highest in enable change (58.3% agreement) and communication (70.9% agreement); and lowest in implementation (58.3% disagreement) and evaluation and research (62.5% disagreement).

**Table 3.** Baseline health promotion competence (*N* = 24).

	Disagree *n* (%)	Neutral *n* (%)	Agree *n* (%)	I don’t know *n* (%)
Enable change	6 (25.0)	2 (8.3)	14 (58.3)	2 (8.3)
Advocate for health	6 (25.0)	4 (16.7)	13 (54.2)	1 (4.2)
Mediate through partnership	6 (25.0)	9 (37.5)	8 (33.3)	1 (4.2)
Communication	4 (16.7)	3 (12.5)	17 (70.8)	
Leadership	7 (29.2)	4 (16.7)	13 (54.2)	
Assessment	12 (50.0)	5 (20.8)	5 (20.8)	2 (8.3)
Planning	12 (50.0)	4 (16.7)	8 (33.3)	
Implementation	14 (58.3)	3 (12.5)	6 (25.0)	1 (4.2)
Evaluation and research	15 (62.5)	3 (12.5)	5 (20.8)	1 (4.2)

### Global citizenship

Table 4 summarizes baseline global citizenship results. The mean score for social responsibility was 4.3. Mean scores for global competence were 3.1 (self-awareness), 4.0 (intercultural communication) and 3.8 (global knowledge). Mean scores for global civic engagement were 3.2 (involvement in civic organizations), 2.8 (political voice) and 3.6 (global civic activism).

**Table 4.** Baseline global citizenship (*N* = 15).

	Mean score
**Social responsibility**	
I think that most people around the world get what they are entitled to have^a^	4.3
It is okay if some people in the world have more opportunities than others^a^	4.1
I think that people around the world get the rewards and punishments they deserve^a^	4.1
In times of scarcity, it is sometimes necessary to use force against others to get what you need^a^	4.1
The world is generally a fair place^a^	4.1
I think that many people around the world are poor because they do not work hard enough^a^	4.9
**Global competence (self-awareness)**	
I know how to develop a place to help mitigate a global environmental or social problem	2.7
I know several ways in which I can make a difference on some of this world’s most worrisome problems	3.1
I am able to get other people to care about global problems that concern me	3.6
**Global competence (intercultural communication)**	
I unconsciously adapt my behaviour and mannerisms when I am interacting with people of other cultures	3.7
I often adapt my communication style to other people’s cultural background	4.0
I am able to communicate in different ways with people from different cultures	4.3
**Global competence (global knowledge)**	
I am informed of current issues that impact international relationships	4.0
I feel comfortable expressing my views regarding a pressing global problem in front of a group of people	4.1
I am able to write an opinion letter to a local media source expressing my concerns over global inequalities and issues	3.3
**Global civic engagement (involvement in civic organizes)**	
Over the next six months, I will participate in a walk, dance, run or bike ride in support of a global cause	3.5
Over the next six months, I plan to get involved with a global humanitarian organize or project	3.1
Over the next six months, I plan to help international people who are in difficulty	3.1
Over the next six months, I plan to get involved in a programme that addresses the global environmental crisis	3.1
Over the next six months, I will work informally with a group toward solving a global humanitarian problem	2.9
Over the next six months, I will pay a membership or make a cash donation to a global charity	3.7
**Global civic engagement (political voice)**	
Over the next six months, I will contact a newspaper or radio to express my concerns about global environmental, social or political problems	2.6
Over the next six months, I will express my views about international politics on a website, blog or chat room	3.0
Over the next six months, I will contact or visit someone in government to seek public action on global issues and concerns	2.7
Over the next six months, I will participate in a campus forum, live music, or theatre performance or other event where young people express their views about global problems	3.1
**Global civic engagement (global civic activism)**	
If at all possible, I will always buy fair-trade or locally grown products and brands	3.9
I will deliberately buy brands and products that are known to be good stewards (caretakers) of marginalized people and places	3.9
I will boycott brands or products that are known to harm marginalized (disempowered) global people and places	2.9

a Negatively worded questions.

### Professional identity

Table 5 summarizes baseline professional identity results. The total score for the sample was 32.4 out of 45.

**Table 5.** Baseline professional identity (*N* = 15).

Social responsibility	Mean score
I feel like I am a member of the health promotion profession	3.1
I feel I have strong ties with members of the health promotion profession	2.9
I find myself making excuses for belonging to the health promotion profession^a^	4.7
I try to hide that I am studying to be part of the health promotion profession^a^	4.7
I am pleased to belong to the health promotion profession	4.5
I can identify positively with members of the health promotion profession	4.1
Being a member of the health promotion profession is important to me	4.1
I feel I share characteristics with other members of the health promotion profession	4.3
Total	32.4

a Negatively worded questions.

### PebblePad usability

Participant SUS scores ranged from 37.5 (F) to 85 (A) with a mean score of 62 (C), indicating average perceived PebblePad usability. Some participants felt comfortable with the platform once they ‘got the hang of it’ and found it ‘user-friendly’; whereas other participants found it ‘really tedious and confusing’ and not overly intuitive.

### Document analysis

Student reflections in 2021 (*n* = 21) and 2022 (*n* = 19) are reported under two domains: 1) perceptions of health promotion knowledge; and 2) development of knowledge and skills within core competencies.

#### Perceptions of health promotion knowledge

Students’ health promotion knowledge varied from ‘holistic and in-depth’ to ‘fundamental aspects’ only. Students indicated that increased knowledge led to better understanding, particularly ‘solidifying concepts such as social justice and human rights’ and involvement in group presentations improved understanding of health promotion models and approaches.

Practical activities also contributed to increased knowledge. Volunteering, ‘contributing to projects’, ‘networking with peers’ and attending industry events influenced this:

‘Working alongside health promotion practitioners [as a volunteer] has helped me gain more knowledge and transferable skills that help me be a good health promotion practitioner.’ (Student, 2022)

#### Development of knowledge and skills within core competencies

First-year health promotion foundations acquired informed knowledge required as practitioners ‘in a real-world setting’ but exposed knowledge gaps, for example:

‘Although I feel I have learnt a sizeable amount, I am not even close to meeting an entry level health promotion standard, which requires a skillset to plan, implement, and evaluate interventions.’ (Student, 2021)

Consistent with survey findings, students reported greatest comfort with communication, predominantly acquired via existing workplace experience or activities such as cultural and diversity training, social media, industry events and reading journal articles. Students not meeting this competency identified lack of workplace experience or working with people from different cultures. Planning, by comparison, was a competency only some students reported meeting, acquired through studies and assessments.

Many students were working toward competencies to advocate for health, enable change and mediate through partnership. Activities supporting these included: volunteering with health organizations, using social media to expand networks, attending health promotion webinars, and becoming a student mentor.

‘I felt empowered. . . as I was able to learn how activities match up to health promotion principles and bring those principles to action’ (Student, 2022)

Students were less likely to report meeting evaluation and research or implementing partnerships with stakeholders domains. Many students indicated these would be subsequently achieved by learning to evaluate health promotion campaigns, developing research skills, contributing to a project and volunteering. Students reporting development of these competencies highlighted volunteering as a research assistant and learning how to use software such as Nvivo and Endnote as activities that contributed to their learning.

Most students indicated that skills to implement effective and efficient, culturally sensitive, and ethical Health Promotion action in partnership with stakeholders would be further developed. Activities identified as contributing to development included completing diversity courses and subsequent implementation of the strategies in their current workplace. No students reported meeting or working toward the assessment competency.

## Discussion

In this paper we presented baseline findings from a cohort study assessing the effect of an updated undergraduate Health Promotion course on first-year student learning. The revised course includes enhanced WIL opportunities (8); self-reflection; competency mapping; and a greater emphasis on global citizenship and professional identity. Changes aim to better prepare students for the workforce through strategies demonstrated to have utility (15). Results provide insights into the first year of the course and implementation of the Passport to Practice initiative.

First-year students reported feeling most confident in communication but least confident in evaluation and research. The initial year serves as a foundation for health promotion values and principles, with subsequent years focusing on competency domains like assessment, implementation, and evaluation and research. Early exposure to the fundamentals reportedly enhanced student understanding of health promotion’s complexity and the role of a health promotion practitioner (16), aligning with established evidence (29). We posit comparing findings with second- and third-year data will help differentiate between ‘entry to practice’ and ‘expert’ competency levels (1).

Summative assessment alone falls short in evaluating competency achievement and fostering reflective practice and professional development, which is vital to the role of a health promotion practitioner (30). Instead, as demonstrated in this study, scaffolding competencies across the course and fostering a sense of progression via sequential and authentic assessment supports competency attainment (31). This also provides students with opportunities to engage with stakeholders, understand ethical principles and develop research and evaluation skills (31).

Findings suggest Passport to Practice positively contributes to professional identity. Using competencies in this context may strengthen professional identity by clarifying the roles and functions of health promotion practitioners (1,32) and encouraging students to examine the broader health promotion sector and their future roles (29). In addition, student cohorts developing and progressing together over time can also strengthen professional identity formation (15).

Experiential learning exposes students to professional health promotion behaviours, strengthening problem-solving and decision-making skills (32). Co-curricular and reflection activities aligned with the Competencies and Standards lay the foundation for continuing professional development requirements for Registered Health Promotion Practitioners (18). Results indicate that students appreciated WIL opportunities and can plan for future activities to address gaps in their competence. This process requires students’ active engagement, mentoring and support from teaching staff (15), which has training and resourcing implications.

Competency-based portfolios assist students to identify further training areas, which the literature suggests is valuable (29). This approach encourages students to reflect across the course rather than within specific subjects/units only (29). PebblePad data, despite feedback recommending improvements, provides early insights into the impact of a personal learning space to document competency achievement. Future results may contribute to literature on e-portfolios and competency-mapping approaches (30).

Equipping students with global citizenship skills prepares them to address complex public health challenges (10). Findings indicate that students excel in the social responsibility dimension, but lag in the political voice category of the global civic engagement dimension. This suggests a need to address local, state, national and global community issues by promoting volunteerism, political activism and community participation (13). Comparing baseline findings with subsequent years after students have completed units with a stronger global civic engagement focus will yield deeper insights.

A study limitation is the small first-year cohort size and low survey response rate, and the potential for student withdrawals before second- and third-year data are collected. There is also the potential for non-response bias due to the possibility that more motivated students responded to the survey request. A mixed-methods design may address this, offering a comprehensive understanding and robust data interpretation (33). A further limitation is the lack of validity and reliability testing on adapted instruments; however, all instruments were piloted with the cohort prior to use. Data triangulation, comparing scores across three timepoints using valid and reliable instruments (13,22,24), combined with qualitative data to explore student perspectives, will increase study reliability and validity (28).

## Conclusion

Assessing a revised Health Promotion course and approach for assessing student achievement of the Competencies and Standards on first-year students’ competence, global citizenship and professional identity provides direction for strengthening health promotion discipline knowledge and skills. Early positive findings will help address knowledge gaps in competency-based health promotion education, and future findings of the cohort study will be used to make recommendations for how the tertiary education sector can engage with industry to strengthen the health promotion workforce.
